# Primary tuberculosis of the parotid gland

**DOI:** 10.1016/S1808-8694(15)30138-5

**Published:** 2015-10-19

**Authors:** Adriano Ulisses Caldart, Cíntia Felício Adriano, Arnoni Ulisses Caldart, Marcos Mocellin

**Affiliations:** a MD. Resident physician in ENT - HC-UFPR; b MD. 3rd year resident physician in ENT - HC-UFPR; c Otorhinolaryngologist -M.S. in Surgery - FURB/UFPR; d PhD. Full Professor of Otorhinolaryngology - UFPR. Head of Otorhinolaryngology HC-UFPR. Paraná Federal University Hospital - Hospital de Clínicas da Universidade Federal do Paraná (HC-UFPR)

**Keywords:** aids, parotid gland, tuberculosis, tumor

## INTRODUCTION

Tuberculosis in the parotid gland is an infectious disease, which manifests itself by an increase in gland volume, makes it lobulated and causes lymphadenitis. It is rare, and literature reports are scarce. With the increase in immunodepressive diseases, especially AIDS, pulmonary involvement is on the rise^1^. Many authors state that, although uncommon, parotid TBC must be part of the differential diagnosis of tumors that increase parotid volume^2^, ^3^. Our goal with the present investigation is to report on a case of primary parotid gland tuberculosis associated with AIDS that mimicked a tumor.

## CASE PRESENTATION

A46 year old man, smoker, harboring the HIV virus, still without clinical treatment, came to our department of otorhinolaryngology complaining of pain and a tumoral mass below the left ear with 5 months of evolution, he did not have fever, had lost some weight and had occasional nighttime sweating.

During his physical examination, we noticed a tough mass, of irregular contour, tender to palpation, of approximately 4 x 3 cm in diameter, in the left parotid region and left side cervical lymphadenomegaly.

He did not have altered lab exams and his chest x-ray was normal. Ultrasound showed a parotid gland with heterogenous echo texture, with ill-defined areas and the involvement of cervical and intraglandular adjacent lymphnodes. CT scan showed parotid asymmetry with a volumetric enlargement of the left side gland, with greater heterogeneity in its upper border, which suggested a mixed tumor with possible malignant transformation and lymphnode infiltration. We did a FNA, which came out negative for neoplastic cells.

Based on this presentation, our initial diagnostic hypothesis matched that of a parotid gland tumor, and we did a partial parotidectomy, with cervical lymphnode excision.

**Figure 1:**
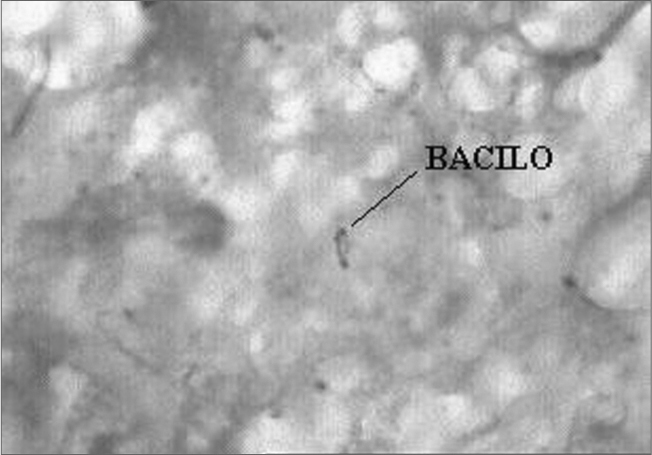
ZIEHL 1000X: Histopathology for BAAR search, showing microorganisms with structures matching those of the Koch’s Bacillus.

The pathology exam showed chronic caseous granulomatous inflammation, matching signs of tuberculosis. Light microscopy with ZIEHL dye showed microorganisms matching the description of Koch’s bacillus (Figure 1).

We then started with chemotherapy for tuberculosis and AIDS. The patient evolved well, with total disease remission. The patient has been under clinical follow up in an outpatient basis for 2 years now, and does not show signs of disease recurrence.

## DISCUSSION

Tuberculosis is a disease that affects mainly young adults, and in most cases, it affects the lungs. In cases when the parotid gland is involved, there usually is a primary pulmonary focus, and blood and lymphatic spread are the most probable causes of secondary infection routes. Ustuner^4^ defined that if a primary disease focus is not found, the infection is clinically called primary tuberculosis of the gland, which can impair diagnosis, since it is very similar to parotid tumors. Holmes^3^ reported that the disease is rare because of an inhibiting effect saliva has over the mycobacterium. In the case reported, we had a young adult, with untreated HIV. Another observation is that in the absence of a prior history of tuberculosis or the finding of a primary focus, as it happened to this patient, the initial diagnostic hypothesis was of a parotid tumor, since this disease, as stated in the literature, mimicked a parotid tumor. Fine needle biopsy did not aid in the diagnosis, and Bhargava^2^ also reported this diagnosis difficult with FNA, because in large neoplasias there are many necrotic areas. Final diagnosis was only possible after the pathology exam, which revealed chronic granulomatous caseous inflammation in an intraglandular lymphnode, matching signs of tuberculosis.

## FINAL REMARKS

Parotid gland tuberculosis, although rare, must be considered as part of the differential diagnosis of tumoral masses in the parotid gland, especially in immunode-pressed patients. Definitive diagnosis can be difficult, but it is important because of the similarities between this disease and neoplasias that affect this organ.
